# The impact of anxiety on vulvar dermatological diseases: a systematic review

**DOI:** 10.1097/JW9.0000000000000282

**Published:** 2026-08-26

**Authors:** Sheila Sharifi, Olivia Katamanin, Richard Moraga, Kelsey S. Flood, Ekene Ezenwa

**Affiliations:** a Georgetown University School of Medicine, Washington, DC; b Chicago Medical School, Rosalind Franklin University, North Chicago, Illinois; c Department of Dermatology, Northwestern University Feinberg School of Medicine, Chicago, Illinois

**Keywords:** Anxiety, mental health, psychodermatology, vulvar dermatitis, vulvar inflammatory dermatoses

## Abstract

**Background:**

Vulvar inflammatory dermatoses, including lichen sclerosus, lichen planus, lichen simplex chronicus, and psoriasis, have been shown to impact mental health and quality of life.

**Objective:**

We aimed to synthesize and evaluate the current literature regarding the association between anxiety and vulvar inflammatory dermatoses.

**Data sources:**

A comprehensive search was conducted using the Ovid MEDLINE, Embase, Cochrane, PubMed, PsycINFO, and Web of Science databases. Search terms included vulvar conditions (eg, “lichen sclerosus,” “lichen planus,” “lichen simplex chronicus,” and “psoriasis”) combined with anxiety-related terms (“anxiety,” “anxious,” and “mental health”).

**Study selection:**

We included appropriate studies (eg, clinical trials, case-control studies, cohort studies, cross-sectional studies, or interview studies) directly addressing vulvar inflammatory dermatoses and anxiety. Studies without quantifiable anxiety outcomes or those that were reviews, protocols, commentaries, or abstracts were excluded. Two reviewers independently screened and resolved discrepancies by consensus.

**Methods:**

A comprehensive search was conducted using the Ovid MEDLINE, Embase, Cochrane, PubMed, PsycINFO, and Web of Science databases. We included appropriate studies (eg, clinical trials, case-control studies, cohort studies, cross-sectional studies, or interview studies) directly addressing vulvar inflammatory dermatoses and anxiety.

**Results:**

Eighteen studies met inclusion criteria. Up to 52% of patients with vulvar inflammatory disease experience symptoms of anxiety that affect sexual functioning, activities of daily living, and plans for pregnancy. The severity of anxiety may be greater in newly diagnosed patients and younger women, while treatment serves as a protective factor. Treatment modalities, including topical corticosteroids, fat grafting, biologics, and platelet-rich plasma, were associated with reductions in anxiety symptoms concurrent with improvement in vulvar disease severity and quality-of-life measures.

**Limitations:**

There is a paucity of data reviewing vulvar dermatoses, specifically vulvar involvement of common dermatologic diseases such as lichen simplex chronicus and psoriasis.

**Conclusion:**

The association between vulvar inflammatory diseases and anxiety may be multifaceted, with emerging evidence suggesting that vulvar disease may increase the risk of anxiety symptoms. Future efforts should focus on establishing targeted interventions that address both the dermatologic and psychological aspects of vulvar disease.

**What is known about this subject in regard to women and their families?**

- Vulvar dermatoses can cause significant psychologic impact in a patient’s quality of life.

**What is new from this article as messages for women and their families?**

- We are continuing to further elucidate the role of anxiety in vulvar dermatologic diseases, noting a potential bidirectional framework of worsening anxiety in the setting of increasing dermatologic disease burden, and vice versa.

## Introduction

Vulvar inflammatory dermatoses encompass a diverse group of chronic skin conditions affecting the vulva, often causing significant discomfort and negatively impacting quality of life. These include lichen sclerosus, lichen planus, lichen simplex chronicus (LSC), and vulvar psoriasis, among others.^1,2^ Vulvar lichen sclerosus (VLS) typically presents with porcelain-white atrophic plaques and may lead to scarring and loss of vulvar architecture.^1,2^ Vulvar lichen planus (VLP) can cause erosive lesions with potential for significant mucosal damage.^1,2^ Lichen simplex chronicus manifests as lichenified, thickened plaques resulting from chronic pruritus.^1,2^ Vulvar psoriasis is characterized by fluctuating exacerbations and remissions, presenting as pruritic, poorly demarcated plaques.^3^ Often difficult to distinguish on physical examination, irritant and allergic contact dermatitis of the vulva both cause pruritus and burning, commonly triggered by external factors such as hygiene products, fabrics, or medications.^4^

The psychological burden of vulvar inflammatory dermatoses remains underexplored. Patients with vulvar disease frequently experience anxiety, depression, and negative self-image, highlighting the complex interplay between dermatologic and mental health conditions.^5,6^ Further, studies suggest that such psychiatric comorbidities may be underdiagnosed due to reluctance and embarrassment to seek care.

While substantial evidence suggests that vulvar inflammatory dermatoses can exacerbate symptoms of anxiety, we suspect a potential bidirectional relationship – where anxiety itself may contribute to the severity and progression of vulvar disease, and vulvar disease may exacerbate preexisting anxiety. In this systematic review, we reviewed and assessed the literature reporting on anxiety in patients with vulvar inflammatory dermatoses, exploring how psychological factors may influence disease outcomes. By synthesizing existing literature, we seek to provide a more comprehensive understanding of this relationship and its implications for patient care.

## Methods

A comprehensive search was conducted using the Ovid MEDLINE, Embase, Cochrane, PubMed, PsycINFO, and Web of Science databases. We used the terms “vulva,” “vulvar,” “vulvas,” “vulvae,” or the MeSH term *Vulva/*, in combination with dermatologic conditions such as “dermatosis,” “dermatitis,” “lichen sclerosus,” “kraurosis,” “lichen planus,” “lichen ruber planus,” “lichen rubra planus,” “lichen planopilaris,” “lichen simplex chronicus,” “neurodermatitis,” “neurodermatitides,” “psoriasis,” or the MeSH terms *Dermatitis/*, *Dermatitis, Atopic/*, *Dermatitis, Contact/*, *Dermatitis, Allergic Contact/*, *Dermatitis, Photoallergic/*, *Dermatitis, Toxicodendron/*, *Dermatitis, Irritant/*, *Dermatitis, Phototoxic/*, *Lichen Planus/*, *Neurodermatitis/*, *Psoriasis/*, and *Vulvar Lichen Sclerosus/*. These were combined with anxiety-related terms including “anxiety,” “anxious,” “mental health,” and the MeSH terms *Anxiety/*, *Anxiety Disorders/*, *Generalized Anxiety Disorder/*, *Anti-Anxiety Agents/*, and *Mental Health/*. After removing duplicate articles, the remaining articles were independently screened and assessed for eligibility by 2 reviewers (S. Sharifi and O. Katamanin/R. Moraga). Abstracts that lacked sufficient information to include or exclude were further assessed using full-text evaluation to determine eligibility. Discrepancies between reviewers were resolved through discussion to reach consensus. Inclusion criteria for this study were: (a) studies must directly address vulvar disease and anxiety, (b) be of an appropriate study type (eg, clinical trials, case-control studies, cohort studies, cross-sectional studies, or interview studies), and (c) be published in peer-reviewed journals in English. Studies were excluded if they were irrelevant to the topic, used an incorrect study type (eg, letters to the editor, commentaries, reviews, conference abstracts, or protocols), were not published in English, or were not published in peer-reviewed journals. Studies lacking quantifiable data regarding anxiety prevalence and characteristics were excluded.

**Fig. 1.**
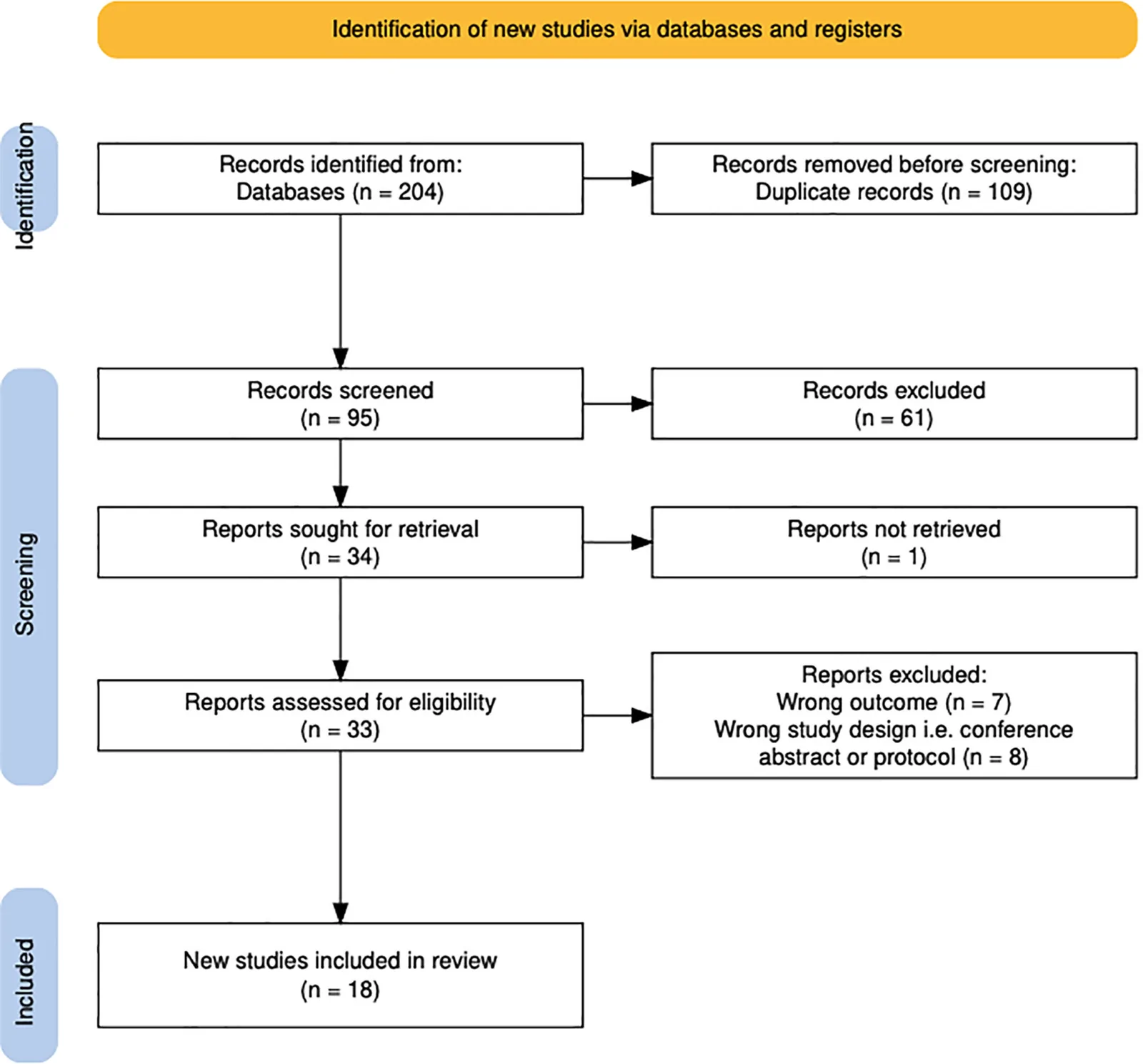
Preferred Reporting Items for Systematic Reviews and Meta-Analyses diagram.

The screening process is outlined in our Preferred Reporting Items for Systematic Reviews and Meta-Analyses diagram (Fig. 1). Following abstract and title screening of 95 studies, 33 underwent full-text review. Fifteen articles were excluded upon full-text review due to study designs that did not meet our search criteria or outcomes that were not relevant to our analysis. Ultimately, 18 studies met the inclusion criteria for this review, with 8 cohort studies, 2 pilot clinical trials, 2 observational studies, 1 case-control study, and 5 cross-sectional studies. The included articles underwent formal quality assessment procedures, using the Joanna Briggs Institute Critical Appraisal Checklists, selected according to study design. Data extraction involved 2 independent reviewers (S. Sharifi and O. Katamanin/R. Moraga) extracting pertinent information from eligible studies and organizing and summarizing the findings, using the online platform, Covidence. A narrative synthesis was conducted to summarize findings (Supplementary Table S1, https://links.lww.com/IJWD/A101).

## Results

### Prevalence and impact of anxiety

Of the 18 studies that met the criteria, 16 included cases of VLS, 6 of VLP, and 3 of LSC. Common validated scales used to assess anxiety were the Hospital Anxiety and Depression Scale, Anxiety (HADS-A), Hamilton Anxiety Rating Scale, Beck Anxiety Inventory, and Vulvar Quality of Life Index. Notably, these tools measure anxiety symptoms and are not diagnostic for generalized anxiety disorder or other anxiety disorders.

A total of 17 of the 18 studies endorsed an association between a diagnosis of vulvar inflammatory disease and anxiety.^7–23^ Recent data has shown that up to 52% of patients with vulvar inflammatory disease had clinically significant anxiety, with multiple studies indicating a higher risk compared to the general population.^13–15^ An electronic health record-based retrospective cohort study of 16,696 patients with VLS compared to matched controls with lichen sclerosus sparing the vulva demonstrated that VLS was associated with an approximately 2-times greater risk of anxiety disorder (3.77% vs 1.96%, relative risk [RR] = 1.93 [1.69–2.20]).^11^ Anxiety is also prevalent in pediatric patients; among 12 pediatric patients with anogenital lichen sclerosus, 75% met the criteria for very high levels of anxiety.^18^

Anxiety secondary to vulvar conditions has been shown to have profound impacts on various aspects of life, including sexual functioning, activities of daily living, and plans for pregnancy.^19,21^ A prospective observational study of 178 patients with VLP and VLS revealed a negative correlation between anxiety and sexual functioning scores, demonstrating increased anxiety associated with worsening sexual functioning, such as a decrease in arousal, desire, orgasm, satisfaction, as well as increased pain.^22^

Research suggests that the time since diagnosis and treatment initiation may play a significant role in the psychological burden experienced by patients. According to a retrospective cohort study of 77 patients with erosive vulvovaginal lichen planus, 29% of women with newly diagnosed disease experienced anxiety, compared to 6% of those with prolonged time since initial diagnosis or treatment with a provider.^10^ Choi *et al*. corroborated these results, demonstrating that VLS patients who received treatment had a decreased risk of anxiety disorder compared to those who were not treated (3.08% vs 4.27%, RR = 0.72 [0.59−0.89]).^11^ Overall, research suggests that over time and with appropriate treatment, anxiety symptoms may ameliorate.

### Impact of disease treatment on anxiety

Several treatment options have been evaluated for their efficacy in reducing anxiety, including topical medications and novel treatments such as lipotransfer and platelet-rich plasma (PRP). Wijaya *et al*. demonstrated in a cross-sectional study of 204 patients with VLS treated with long-term topical corticosteroids that anxiety scores were significantly lower on the VLQI compared with pretreatment levels (median = 0 [interquartile range {IQR} = 0.0–0.3] vs 0.8 [IQR = 1.1–2.0]; *P* < 0.001).^21^ The same research group found that patients who reported a poorer quality of life during long-term topical corticosteroid treatment tended to have higher levels of anxiety, as well as concerns related to sexuality and body image (1.3 [IQR = 1.0–1.5]).^23^ Notably, these patients with elevated anxiety scores also demonstrated lower adherence to topical corticosteroids and had more active disease, including scarring progression (mean rank = 94.8 vs 66.6; *P* = 0.02), unstable disease (mean rank = 94.3 vs 65.5; *P* = 0.005), and urinary incontinence (mean rank = 82.0 vs 65.5; *P* = 0.04), suggesting that treatment adherence may influence both disease severity and associated anxiety.^21^

Novel therapies for vulvar inflammatory dermatoses are emerging, though evidence is limited to small cohort studies. Almadori *et al*. examined fat grafting in their prospective cohort of 33 patients with VLS not responsive to topical corticosteroids and reported statistically significant improvements in anxiety scores following fat grafting (*P* < 0.0001).^7^ Boero *et al*. supported these findings in a larger retrospective cohort study of 71 patients, reporting that at follow-up (2–11 years) after fat grafting for vulvar VLS, patients had improved HADS-A scores (*P* < 0.029).^8^ In both studies, fat grafting was associated with reported improvement in vulvar disease features, including fibrosis and scarring, with concurrent reductions in anxiety scores; however, these findings were derived from relatively small cohorts.^7,8^ Patients treated with tildrakizumab, a biologic inhibiting the interleukin-23 (IL-23) receptor, had statistically significantly decreased VLQI scores in the anxiety domain (4.04, 95% confidence interval [CI] = 2.38–5.78) compared to patients treated with traditional topical (6.69, 95% CI = 5.02–8.38; *P* = 0.006) or systemic (6.58, 95% CI = 4.81–8.35; *P* < 0.001) modalities.^16^ Finally, autologous PRP was examined in a pilot study of 50 patients, with median anxiety scores significantly decreasing after 3 monthly vulvar injections (*P* < 0.001).^9^ It is worth noting that these therapies also demonstrated clinical improvement, which may contribute to the observed reductions in anxiety.^9,16^

According to a prospective observational study of 186 female patients with nonneoplastic epithelial disorders of the vulva (including VLS, VLP, and LSC), anxiety was a negative prognostic factor for treatment outcomes with the use of high-intensity focused ultrasound, a novel physiotherapy technique.^12^ Hence, while conventional and emerging therapies have demonstrated the potential to improve clinical symptoms and disease-related anxiety, anxiety itself may correlate with treatment outcomes.^13^

## Discussion

Our results indicate a significant association between vulvar inflammatory dermatoses and anxiety, with implications regarding comorbid disease progression and severity. While causality cannot be established, it is plausible that these effects exist in a bidirectional relationship, in which vulvar disease may increase the risk of anxiety, while preexisting anxiety may, in turn, contribute to symptom amplification, delayed clinical improvement, or persistent symptom burden despite appropriate therapy. Similar bidirectional patterns have been observed in other inflammatory dermatoses, such as chronic urticaria.^24^ Conversely, the unpredictable nature and visibility of the disease may contribute to psychological distress.^24^ Psoriasis demonstrates a comparable cycle, in which the disease is associated with increased rates of anxiety and depression, independent of disease severity. At the same time, psychological stress is also a known trigger for psoriasis flares, likely mediated via the HPA axis and proinflammatory cytokines such as tumor necrosis factor alpha and IL-17, perpetuating a vicious cycle between psoriasis and mental health burden.^25^

Importantly, worsening patient-reported symptoms in vulvar disease may not uniformly reflect objective progression of inflammatory disease. Anxiety has been shown to heighten symptom perception through mechanisms such as central sensitization, attentional bias, and an overlap between anxiety and somatic symptom reporting.^26,27^ In this context, symptoms may feel more severe without corresponding changes in clinical disease activity. This distinction underscores the need to interpret anxiety-associated symptom burden not solely as disease worsening, but as an interaction between peripheral inflammation and central perceptual processing.

Anxiety may be particularly elevated in patients with newly diagnosed vulvar conditions.^10^ These psychological effects may be more pronounced in certain populations, such as premenopausal women.^11^ Not only does anxiety peak in the young adult years, but also, younger women may have greater concerns over fertility, body image, and sexual functioning, heightening the psychological burden of vulvar disease.^17,28^ Moreover, fluctuations in anxiety symptoms throughout the female lifespan are also postulated to be secondary to hormonal changes.^29^ Future research should explore the interplay between hormonal factors, vulvar disease severity, and psychological risks throughout varied age groups.

Treatment stands as a protective factor, with a greater risk of anxiety in those left untreated.^11^ First-line management with topical steroids remains the cornerstone of therapy and is essential not only for disease control, but also to mitigate psychosocial distress as higher anxiety levels are associated with inadequate or suboptimal management. Several alternative treatment modalities, including fat grafting, biologics, and PRP, have shown improvement in reducing anxiety symptoms, with fat grafting reporting robust results within small participant studies.^7,8,30^ Nonetheless, some women continue to experience mood changes and sexual dysfunction despite vulvar symptom improvement, indicating the long-term emotional impact of the disease.^23,31^

Beyond symptomatic improvement, emerging evidence suggests that inflammatory modulation may influence anxiety-related pathways. Biologic agents such as tildrakizumab, which target the IL-23 axis, have been associated with improvements in anxiety-related quality-of-life domains among patients with vulvar inflammatory disease.^16^ These observations align with broader psychoneuroimmunology literature demonstrating associations between peripheral inflammatory cytokines and anxiety symptoms.^32^ While these agents are currently not indicated for the treatment of anxiety, these findings underscore the interconnectedness of immune signaling, chronic inflammation, and psychological distress, particularly in inflammatory dermatoses affecting sensitive anatomical sites.

Patients often express concern about long-term health implications and sexual functioning, underscoring the benefit of physician counseling and integrative treatment approaches.^21^ Further, anxiety relating to the risk of cancer in cases of VLS emphasizes the importance of education at the time of diagnosis.^21^ Holistic treatment may improve outcomes, including pelvic floor therapy, cognitive behavioral therapy, and social support groups.^33^ The utility of these treatment modalities, as well as anti-anxiety medications, remains underexplored and warrants further investigation. Importantly, the vulvar dermatologic education of dermatology and obstetrics and gynecology trainees remains limited, suggesting an opportunity to improve care delivery through increased interdisciplinary collaboration.^34^

Our review is subject to several limitations that necessitate consideration. First, the reported prevalence of anxiety varied across studies, potentially due to differences in diagnostic criteria. Furthermore, geographic and demographic influences were not directly addressed in our analysis, potentially impacting the generalizability of results. Notably, our review included only 3 articles addressing LSC, and none for vulvar psoriasis. This paucity of data limits our ability to fully characterize the relationship between specific vulvar conditions and anxiety. Finally, individual factors such as cultural preferences and socioeconomic status must be considered when assessing psychological impact. Future studies should use larger, more diverse cohorts and longitudinal designs with standardized screening tools to better quantify the relationship between vulvar inflammatory dermatoses and anxiety.

## Conclusion

This systematic review purports the increasingly evident bidirectional relationship between anxiety and vulvar inflammatory dermatoses, highlighting the prevalence of anxiety in inflammatory dermatoses and, therefore, the role for a multidisciplinary approach to patient care for patients with vulvar conditions. Anxiety may not only exacerbate disease symptoms but also influence treatment outcomes and overall quality of life. Given the psychological burden associated with vulvar disease, there is benefit in integrating mental health support alongside dermatologic and gynecologic care. Future research could focus on refining long-term treatment approaches, exploring the role of hormonal influences, and identifying targeted interventions that address both the dermatologic and psychological aspects of vulvar disease.

## Conflicts of interest

The authors made the following disclosures: KSF is an investigator for Abbvie, Moonlake, and Novartis. She is a consultant for Leo Pharma. She is on the board of directors for the Vulvar Disease Research Consortium. She previously received fellowship funding from Abbvie, the National Psoriasis Foundation, and Janssen Biotech, Inc, which was paid directly to her institution. EE is a consultant for Leo Pharma. She is on the board of directors for the Vulvar Disease Research Consortium.

## Funding

None.

## Study Approval

Because this systematic review utilizes existing literature and does not collect primary data from human participants, it did not require institutional review board approval.

## Author contributions

SS: Participated in conceptualization, data extraction, writing, and editing. OK: Participated in data extraction, writing, and editing. RM: Participated in data extraction, writing, and editing. KSF: Participated in supervision, writing, and editing. EE: Participated in supervision, writing, and editing.

## Supplementary data

Supplementary material associated with this article can be found at https://links.lww.com/IJWD/A101.

## Acknowledgements

We thank C. Scott Dorris at the Georgetown University Library for his contributions to the manuscript preparation.

## Supplementary Material



## References

[1] Simonetta C Burns EK Guo MA. Vulvar dermatoses: a review and update. Mo Med 2015;112:301–7. PMC6170060 26455062

[2] Prabhu S Krishna S. Vulvar inflammatory disorders: a review. JSSTD 2022;4:188–95. doi: 10.25259/jsstd_11_2021.

[3] Wu M Fischer G. Understanding and managing vulvar psoriasis in girls: findings from a cohort study. Pediatr Dermatol 2025;42:273–7. doi: 10.1111/pde.15816. 10.1111/pde.15816 39592125

[4] Bauer A Rödiger C Greif C Kaatz M Elsner P. Vulvar dermatoses – irritant and allergic contact dermatitis of the vulva. Dermatology 2005;210:143–9. doi: 10.1159/000082570. 10.1159/000082570 15724097

[5] Jabłonowska O Woźniacka A Szkarłat S Żebrowska A. Female genital lichen sclerosus is connected with a higher depression rate, decreased sexual quality of life and diminished work productivity. PLoS One 2023;18:e0284948. doi: 10.1371/journal.pone.0284948. 10.1371/journal.pone.0284948 PMC10128971 37098076

[6] Meyer-Wilmes P Wittenborn J Kupec T Caspers R Stickeler E Iborra S. Patient satisfaction and sexual issues in vulvar lichen sclerosus treatment: a monocentric certified dysplasia unit survey analysis. Arch Gynecol Obstet 2024;310:507–13. doi: 10.1007/s00404-024-07519-w. 10.1007/s00404-024-07519-w PMC11169031 38703281

[7] Almadori A Hansen E Boyle D. Fat grafting improves fibrosis and scarring in vulvar lichen sclerosus: results from a prospective cohort study. J Low Genit Tract Dis 2020;24:305–10. doi: 10.1097/LGT.0000000000000520. 10.1097/LGT.0000000000000520 32205767

[8] Boero V Brambilla M Di Loreto E. Fat grafting in vulvar lichen sclerosus: long term follow-up. J Low Genit Tract Dis 2023;27:365–72. doi: 10.1097/LGT.0000000000000766. 10.1097/LGT.0000000000000766 37551790

[9] Boero V Cetera GE Caia C. Is there a role for platelet rich plasma injection in vulvar lichen sclerosus? A self-controlled pilot study. Arch Gynecol Obstet 2024;309:2719–26. doi: 10.1007/s00404-024-07424-2. 10.1007/s00404-024-07424-2 38523203

[10] Cheng H Oakley A Conaglen JV Conaglen HM. Quality of life and sexual distress in women with erosive vulvovaginal lichen planus. J Low Genit Tract Dis 2017;21:145–9. doi: 10.1097/LGT.0000000000000282. 10.1097/LGT.0000000000000282 27906807

[11] Choi UE Nicholson RC Agrawal P. Involvement of vulva in lichen sclerosus increases the risk of antidepressant and benzodiazepine prescriptions for psychiatric disorder diagnoses. Int J Impot Res 2024;36:641–6. doi: 10.1038/s41443-023-00793-3. 10.1038/s41443-023-00793-3 37973860

[12] Feng T Wang L Zhu D. Factors influencing the clinical efficacy of high-intensity focused ultrasound in the treatment of non-neoplastic epithelial disorders of the vulva: a retrospective observational study. Int J Hyperthermia 2021;38:1457–61. doi: 10.1080/02656736.2021.1985628. 10.1080/02656736.2021.1985628 34620032

[13] Grasso F Grasso V. Anxiety as risk factor for vulvar lichen sclerosus et atrophicus. Gazz Med Ital Arch Sci Med 2014;173:317–9.

[14] Hickey S Bell H. Quality of life in the vulvar clinic: a pilot study. J Low Genit Tract Dis 2010;14:225–9. doi: 10.1097/LGT.0b013e3181dc1e45. 10.1097/LGT.0b013e3181dc1e45 20592559

[15] Kelekçi KH Özyurt S Özkan B Karaca S Karakuzu A Bilgin I. The impact of inflammatory and infectious diseases of vulvar on quality of life. J Menopausal Med 2016;22:131–8. doi: 10.6118/jmm.2016.22.3.131. 10.6118/jmm.2016.22.3.131 PMC5256362 28119892

[16] Kherlopian A Fischer G. Comparing quality of life in women with vulvovaginal lichen planus treated with topical and systemic treatments using the vulvar quality of life index. Australas J Dermatol 2023;64:e125–34. doi: 10.1111/ajd.14032. 10.1111/ajd.14032 37036241

[17] Kolitz E Gammon L Mauskar M. Vulvar lichen sclerosus in women of reproductive age. Proc (Bayl Univ Med Cent) 2021;34:349–51. doi: 10.1080/08998280.2021.1885093. 10.1080/08998280.2021.1885093 PMC8059896 33953458

[18] Osminina MK Khachatryan LG Podchernyayeva NS. Clinical and psychological features of children with anogenital lichen sclerosus: approaches to the choice of therapy. New Armenian Med J 2020;14:88–99.

[19] Shaffer AB Cigna ST Pope R Krapf JM. Pregnancy, parturition and postpartum considerations among patients with vulvar lichen sclerosus: a retrospective cross-sectional online survey. BJOG 2024;131:327–33. doi: 10.1111/1471-0528.17601. 10.1111/1471-0528.17601 37424180

[20] Wijaya M Lee G Fischer G Lee A. Quality of life in vulvar lichen sclerosus patients treated with long-term topical corticosteroids. J Low Genit Tract Dis 2021;25:158–65. doi: 10.1097/lgt.0000000000000599. 10.1097/LGT.0000000000000599 33746196

[21] Wijaya M Lee G Fischer G. Quality of life of women with untreated vulval lichen sclerosus assessed with vulval quality of life index (VQLI). Australas J Dermatol 2021;62:177–82. doi: 10.1111/ajd.13530. 10.1111/ajd.13530 33508152

[22] Yildiz S Cengiz H Kaya C. Evaluation of genital self-image and sexual dysfunction in women with vulvar lichen planus or lichen sclerosus. J Psychosom Obstet Gynaecol 2022;43:99–106. doi: 10.1080/0167482x.2020.1857359. 10.1080/0167482X.2020.1857359 33297796

[23] Wijaya M Lee G Fischer G. Why do some patients with vulval lichen sclerosus on long-term topical corticosteroid treatment experience ongoing poor quality of life? Australas J Dermatol 2022;63:463–72. doi: 10.1111/ajd.13926. 10.1111/ajd.13926 PMC9828553 36208206

[24] Staubach P Eckhardt-Henn A Dechene M. Quality of life in patients with chronic urticaria is differentially impaired and determined by psychiatric comorbidity. Br J Dermatol 2006;154:294–8. doi: 10.1111/j.1365-2133.2005.06976.x. 10.1111/j.1365-2133.2005.06976.x 16433799

[25] Chu M Shen S Zhu Z. Association of psoriasis with depression, anxiety, and suicidality: a bidirectional two-sample Mendelian randomization study. J Dermatol 2023;50:1629–34. doi: 10.1111/1346-8138.16941. 10.1111/1346-8138.16941 37697936

[26] Crombez G Van Damme S Eccleston C. Hypervigilance to pain: an experimental and clinical analysis. Pain 2005;116:4–7. doi: 10.1016/j.pain.2005.03.035. 10.1016/j.pain.2005.03.035 15927387

[27] Woolf CJ. Central sensitization: implications for the diagnosis and treatment of pain. Pain 2011;152 3 Suppl:S2–S15. doi: 10.1016/j.pain.2010.09.030. 10.1016/j.pain.2010.09.030 PMC3268359 20961685

[28] Goodwin RD Weinberger AH Kim JH Wu M Galea S. Trends in anxiety among adults in the United States, 2008-2018: rapid increases among young adults. J Psychiatr Res 2020;130:441–6. doi: 10.1016/j.jpsychires.2020.08.014. 10.1016/j.jpsychires.2020.08.014 PMC7441973 32905958

[29] Hantsoo L Epperson CN. Anxiety disorders among women: a female lifespan approach. Focus (Am Psychiatr Publ) 2017;15:162–72. doi: 10.1176/appi.focus.20160042. 10.1176/appi.focus.20160042 PMC5613977 28966563

[30] Boero V Caia C Cetera GE. First use of cord blood platelet-rich plasma in the treatment of vulvar lichen sclerosus: a preliminary study towards a randomized controlled trial. Blood Transfus 2025;23:348–56. doi: 10.2450/BloodTransfus.875. 10.2450/BloodTransfus.875 PMC12274200 39804749

[31] Burrows LJ Creasey A Goldstein AT. The treatment of vulvar lichen sclerosus and female sexual dysfunction. J Sex Med 2011;8:219–22. doi: 10.1111/j.1743-6109.2010.02077.x. 10.1111/j.1743-6109.2010.02077.x 20955314

[32] Costello H Gould RL Abrol E Howard R. Systematic review and meta-analysis of the association between peripheral inflammatory cytokines and generalised anxiety disorder. BMJ Open 2019;9:e027925. doi: 10.1136/bmjopen-2018-027925. 10.1136/bmjopen-2018-027925 PMC6661660 31326932

[33] Sivalingam V Tamber K. Understanding the impact of life with vulval lichen sclerosus. Br J Dermatol 2022;187:840. doi: 10.1111/bjd.21846. 10.1111/bjd.21846 PMC10087758 36052748

[34] Comstock JR Endo JO Kornik RI. Adequacy of dermatology and ob-gyn graduate medical education for inflammatory vulvovaginal skin disease: a nationwide needs assessment survey. Int J Womens Dermatol 2020;6:182–5. doi: 10.1016/j.ijwd.2020.01.008. 10.1016/j.ijwd.2020.01.008 PMC7330429 32637541

