# Supplementary material for: The impact of anxiety on vulvar dermatological diseases: a systematic review
Source: Int J Womens Dermatol. 2026 Aug 26;12(3):e282. doi: 10.1097/JW9.0000000000000282 (PMC13521501; doi:10.1097/JW9.0000000000000282)
Supplement: Supplementary file 1 [file jw9-12-e282-s001.docx]

**Supplemental Table 1. Characteristics of Included Studies**

| **Title of Study** | **Type of Study/Population** | **Vulvar Disease State Studied** | **Tools Used to Measure Anxiety** | **Key Findings** |
| --- | --- | --- | --- | --- |
| Fat grafting improves fibrosis and scarring in vulvar lichen sclerosus: Results from a prospective cohort study.[^7^](https://paperpile.com/c/2y7hMO/ql5K) | Prospective cohort study of 33 patients | VLS | HADS-A | - Pretreatment median HADS-A score was 11.8 - Fat grafting was associated with a significant improvement in anxiety levels to 8.1 (p<.0001) |
| Fat grafting in vulvar lichen sclerosus: Long term follow-up.[^8^](https://paperpile.com/c/2y7hMO/hhr6) | Retrospective cohort study of 71 patients | VLS | HADS-A | - Within 2-10 years follow-up after fat grafting, patients’ HADS-A score decreased from 14.1% to 7% (p<0.029) |
| First use of cord blood platelet-rich plasma in the treatment of vulvar lichen sclerosus: a preliminary study towards a randomized controlled trial.[^34^](https://paperpile.com/c/2y7hMO/9dd08) | Prospective, single arm pilot study of 10 patients | VLS | HADS-A | - Median HADS-A before treatment with three vulvar CB-PRP injections monthly and following treatment was unchanged [4.0, 4.0] (p=.188) |
| Is there a role for platelet rich plasma injection in vulvar lichen sclerosus? A self-controlled pilot study.[^9^](https://paperpile.com/c/2y7hMO/yTfu) | Prospective, single arm pilot study of 50 patients treated with CB-PRP | VLS | HADS-A | - Median HADS-A before treatment with three vulvar CB-PRP injections monthly was 7.0 [5.0–11.0] - At six months following the last injection, the median was 5.0 [3.0–8.0] (p<.001) |
| Quality of life and sexual distress in women with erosive vulvovaginal lichen planus.[^10^](https://paperpile.com/c/2y7hMO/7flbH) | Prospective observational study of 77 patients | VLS (n=48), EVLP (n=17), vulvar dermatitis (n=12) | HADS-A | - 16% of all patients had anxiety based on the HADS-A score - 29% of women with newly diagnosed vulvar dermatoses experienced anxiety, compared to 6% of those who had been diagnosed for longer and/or underwent treatment |
| Involvement of vulva in lichen sclerosus increases the risk of antidepressant and benzodiazepine prescriptions for psychiatric disorder diagnoses.[^11^](https://paperpile.com/c/2y7hMO/WdB1) | Retrospective cohort study of 16,696 patients with VLS with matched controls | VLS | HADS-A | - VLS was associated with an increased risk of anxiety disorder (3.77% vs. 1.96%, RR 1.93 [1.69–2.20]) compared to women with lichen sclerosus that spared the vulva - When stratified by age, VLS was associated with an increased risk of new anxiety disorder in premenopausal women (age 18-40) (RR 2.35 [1.35–4.10]), perimenopausal women (RR 2.23 [1.46–3.41]), and postmenopausal women (RR 1.82 [1.58–2.09]) - Those who received VLS treatment had a decreased risk of anxiety disorder (3.08% vs. 4.27%, RR 0.72 [0.59−0.89]) compared to those who did not receive treatment |
| Factors influencing the clinical efficacy of high-intensity focused ultrasound in the treatment of non-neoplastic disorders of the vulva: a retrospective observational study.[^12^](https://paperpile.com/c/2y7hMO/QXEO) | Retrospective observational study of 186 patients | Non-neoplastic epithelial disorders of the vulva, including VLS, LSC, and VLP | NR | - Anxiety was a poor prognostic factor for effectiveness of HIFU |
| Anxiety as risk factor for vulvar lichen sclerosus et atrophicus.[^13^](https://paperpile.com/c/2y7hMO/zj7g) | Retrospective observational study of 25 patients | VLS | HAM-A | - Among patients with VLS, there was a significantly higher incidence of moderate and severe anxiety compared to controls (p=.001 and p=.029, respectively) |
| Quality of life in the vulvar clinic: a pilot study.[^14^](https://paperpile.com/c/2y7hMO/TbAI) | Prospective cohort study of 23 patients | Eczema/LSC, VLS, VLP, vulvodynia | HADS-A | - HADS-A scores were obtained at initial consultation in a multidisciplinary vulvar clinic - 52% of patients scored 8 or higher on the HADS-A score, indicating clinically significant anxiety |
| The Impact of Inflammatory and Infectious Diseases of Vulvar on Quality of Life.[^15^](https://paperpile.com/c/2y7hMO/T94P) | Cross-sectional survey of 60 patients with vulvar dermatoses, 60 patients with vulvar infections and 60 matched controls | Vulvar dermatoses and vulvar infectious diseases | HAM-A | - Average HAM-A score for vulvar dermatoses patients was 10.06 out of 21, compared to 7.933 in healthy controls (p=.035) |
| Comparing quality of life in women with vulvovaginal lichen planus treated with topical and systemic treatments using the vulvar quality of life index.[^16^](https://paperpile.com/c/2y7hMO/TRpZ) | Retrospective cohort study of 112 patients | VLP | VQLI | - At baseline, 13.4% of patients had anxiety/depression - Patients escalated to treatment with tildrakizumab demonstrated statistically significant decreased mean VQLI scores for anxiety (4.04, 95% CI 2.38–5.78) compared to when they were treated with either topical (6.69, 95% CI 5.02–8.38; p=.006) or systemic (6.58, 95% CI 4.81–8.35; p<.001) treatments |
| Vulvar lichen sclerosus in women of reproductive age.[^17^](https://paperpile.com/c/2y7hMO/Fi6f) | Retrospective cohort study of 33 patients | VLS | NR | - 21% of patients with VLS had comorbid depression or anxiety - 1 patient endorsed worsening depression secondary to sexual dysfunction |
| Clinical and psychological features of children with anogenital lichen sclerosus: approaches to the choice of therapy.[^18^](https://paperpile.com/c/2y7hMO/eblZv) | Retrospective cohort study of 12 patients | Anogenital lichen sclerosus, female (n=11) and male (n=1) | CMAS (if aged > 8 years) or “Choose the right face” (if <8 years) | - Before treatments, patients scored very high (75%), significantly increased (16.7%) and slightly increased (8.3%) anxiety levels - After 12 months of stepwise therapy with topical corticosteroids and calcineurin inhibitors, followed by oral immunosuppressants, if needed, 50% of the children had a decrease to insignificant levels of anxiety, with 83% of children demonstrating improvement; no statistical analysis available |
| Pregnancy, parturition and postpartum considerations among patients with vulvar lichen sclerosus: A retrospective cross-sectional online survey.[^19^](https://paperpile.com/c/2y7hMO/zEYF) | Cross-sectional study of 134 patients | VLS | NR | - 50% of pregnant patients with VLS reported anxiety about delivery |
| Quality of life in vulvar lichen sclerosus patients treated with long-term topical corticosteroids.[^20^](https://paperpile.com/c/2y7hMO/CW7bU) | Prospective cross-sectional study of 204 patients | VLS, pretreatment (n=68) and long-term treatment (n=136) | VQLI | - Treatment with long-term corticosteroids resulted in significantly lower anxiety (p<.001) - Within the treatment group, elevated anxiety levels were significantly associated with scarring progression (p = .02), unstable disease course (p = .005), and the presence of urinary incontinence (p = .04) - Those with poor disease control exhibited significantly higher anxiety levels compared to those with well-controlled disease (p = .03) |
| Quality of life of women with untreated vulval lichen sclerosus assessed with vulval quality of life index (VQLI).[^21^](https://paperpile.com/c/2y7hMO/6Uoc) | Prospective cross-sectional study of 68 patients | VLS, untreated | VQLI | - 19% of patients with untreated VLS had a past medical diagnosis of anxiety and/or depression - Sexuality showed the strongest correlation with anxiety (p<0.001) - Anxiety was most strongly correlated with activities of daily living (p<0.001) - Within the questions on anxiety, worries about long-term health implications was the highest-scoring item |
| Evaluation of genital self-image and sexual dysfunction in women with vulvar lichen planus or lichen sclerosus.[^22^](https://paperpile.com/c/2y7hMO/F6c1) | Prospective observational study of 178 patients | VLP (n=21), VLS (n=59), fungal vulvitis (n= 48) | BAI | - VLP and VLS patients had significantly higher BAI scores compared to fungal vulvitis and healthy controls (p<.001) - A negative correlation was observed between BAI scores and FSFI total and sub scores in VLP and VLS patients (p<.005) indicating that patients with higher anxiety (BAI scores) had lower sexual functioning (FSFI score) |
| Why do some  patients with  vulval lichen  sclerosus on  long-term  corticosteroid  treatment  experience  ongoing poor quality of life?^23^ | Prospective cross-  sectional study of  255 patients | VLS, pre-  treatment  (n=67) and  long-term  treatment  (n=188) | VQLI | - Anxiety symptoms were significantly lower in patients with full treatment adherence and optimal disease control (p<.05 and p<.001, respectively) - In patients with ongoing poor QOL on long-term corticosteroids, the two greatest areas of concern were sexuality and anxiety |

**Table Legend**

VLS: Vulvar lichen sclerosus

HADS-A: Hospital Anxiety and Depression Scale, Anxiety

CB-PRP: Cord blood platelet-rich plasma

EVLP: Erosive vulvovaginal lichen planus

LSC: Lichen simplex chronicus

VLP: Vulvar lichen planus

NR: Not Recorded

VQLI: Vulvar Quality of Life Index

HAM-A: Hamilton Anxiety Rating Scale

LSA: Lichen sclerosus et atrophicus

CMAS: Children's Form of Manifest Anxiety Scale

BAI: Beck Anxiety Index

FSFI: Female Sexual Function Index
